# A robust and reliable non-invasive test for stress responsivity in mice

**DOI:** 10.3389/fnbeh.2014.00125

**Published:** 2014-04-15

**Authors:** Annemarie Zimprich, Lillian Garrett, Jan M. Deussing, Carsten T. Wotjak, Helmut Fuchs, Valerie Gailus-Durner, Martin Hrabě de Angelis, Wolfgang Wurst, Sabine M. Hölter

**Affiliations:** ^1^Helmholtz Zentrum München, German Research Center for Environmental Health, Institute of Developmental GeneticsNeuherberg, Germany; ^2^German Mouse Clinic, Helmholtz Zentrum MünchenNeuherberg, Germany; ^3^Max Planck Institute of PsychiatryMunich, Germany; ^4^Helmholtz Zentrum München, German Research Center for Environmental Health, Institute of Experimental GeneticsNeuherberg, Germany; ^5^Lehrstuhl für Experimentelle Genetik, Technische Universität MünchenMünchen, Germany; ^6^German Center for Diabetes Research (DZD)Neuherberg, Germany; ^7^Lehrstuhl für Entwicklungsgenetik, Technische Universität MünchenMünchen, Germany; ^8^Deutsches Zentrum für Neurodegenerative Erkrankungen e. V.Munich, Germany; ^9^Munich Cluster for Systems NeurologyMünchen, Germany

**Keywords:** Open Field test, acute restraint stress, mouse mutants, behavioral read-out, hyperlocomotion, corticosterone

## Abstract

Stress and an altered stress response have been associated with many multifactorial diseases, such as psychiatric disorders or neurodegenerative diseases. As currently mouse mutants for each single gene are generated and phenotyped in a large-scale manner, it seems advisable also to test these mutants for alterations in their stress responses. Here we present the determinants of a robust and reliable non-invasive test for stress-responsivity in mice. Stress is applied through restraining the mice in tubes and recording behavior in the Open Field 20 min after cessation of the stress. Two hours, but not 15 or 50 min of restraint lead to a robust and reproducible increase in distance traveled and number of rearings during the first 5 min in the Open Field in C57BL/6 mice. This behavioral response is blocked by the corticosterone synthesis inhibitor metyrapone, but not by RU486 treatment, indicating that it depends on corticosteroid secretion, but is not mediated via the glucocorticoid receptor type II. We assumed that with a stress duration of 15 min one could detect hyper-responsivity, and with a stress duration of 2 h hypo-responsivity in mutant mouse lines. This was validated with two mutant lines known to show opposing effects on corticosterone secretion after stress exposure, corticotropin-releasing hormone (CRH) over-expressing mice and CRH receptor 1 knockout (KO) mice. Both lines showed the expected phenotype, i.e., increased stress responsivity in the CRH over-expressing mouse line (after 15 min restraint stress) and decreased stress responsivity in the CRHR1-KO mouse line (after 2 h of restraint stress). It is possible to repeat the acute stress test several times without the stressed animal adapting to it, and the behavioral response can be robustly evoked at different ages, in both sexes and in different mouse strains. Thus, locomotor and rearing behavior in the Open Field after an acute stress challenge can be used as reliable, non-invasive indicators of stress responsivity and corticosterone secretion in mice.

## Introduction

Stress is a major risk-factor in many multifactorial diseases, such as cardiovascular diseases, psychiatric disorders like anxiety and depression, as well as neurodegenerative diseases such as Alzheimer's and Parkinson's disease (Lupien et al., 1994; Black and Garbutt, 2002; Esch et al., 2002; Bunker et al., 2003; De Kloet et al., 2005; Sotiropoulos et al., 2008; Catania et al., 2009). Still the etiology of these diseases remains elusive, as the interplay between genetic as well as environmental factors is difficult to disentangle. Most of our knowledge about the impact of stress on a disease is derived from the research field of anxiety and depression. Severe stress can trigger depression and it is correlated with the onset and recurrence of depressive episodes (Bao et al., 2008; Pittenberger and Duman, 2008; Sandi and Richter-Levin, 2009).

Exposure to a stressor activates the hypothalamic-pituitary-adrenal (HPA)-axis, by secretion of corticotropin-releasing hormone (CRH, aka CRF) and vasopressin from the paraventricular nucleus of the hypothalamus at the level of the median eminence (for review see Stratakis and Chrousos, 1995; Tsigos and Chrousos, 2002; De Kloet et al., 2005). Both neuropeptides in concert lead to the release of adrenocorticotropic hormone (ACTH) from the anterior pituitary into the circulation. Via the blood stream ACTH reaches the adrenals atop of the kidneys, where corticosteroids (CORT; cortisol in humans, corticosterone in rodents) are synthesized and secreted in its response. CORT, a steroid hormone, reaches many target tissues throughout the entire body and feeds-back on several parts of the brain. It exerts its negative feedback at the level of the pituitary, the hypothalamus and the hippocampus, which leads to a shut-down of the stress-response. CORT has two major receptors, the glucocorticoid receptor (GR) and the mineralocorticoid receptor (MR). Both receptors are distributed differentially throughout the brain; the MR is mainly expressed in limbic structures, whereas the GR is expressed widely throughout the entire brain, i.e., in subcortical (e.g., paraventricular nucleus and hippocampus) and cortical structures (e.g., prefrontal cortex) as well as in the brain stem (Reul and De Kloet, 1985). The MR, with its 6–10 times higher affinity for CORT than GR, is mainly involved in the control of diurnal CORT secretion patterns, and the GR plays a role during peak secretions before waking and during stress (Reul and De Kloet, 1985; De Kloet et al., 1999).

The correct functioning of the HPA-axis in response to a stressor is vital for an organism. If this system is out of equilibrium, devastating consequences can occur. An imbalance of the HPA-axis is seen in patients with major depression, anxiety-disorders and Cushing's syndrome only to mention a few (Brown et al., 1999; Pomara et al., 2003). Still it is debated whether an HPA-axis hyperactivity is the cause or the consequence in depression (Neigh and Nemeroff, 2006). Nevertheless, it seems clear that an increased stress responsivity can be the cause of increased vulnerability to stress associated diseases (Pardon and Rattray, 2008; Sandi and Richter-Levin, 2009).

To understand the genetic contribution for underlying pathological mechanisms in human disease, mouse models for every gene are generated and these mice are subsequently phenotyped in a standardized large-scale manner. Projects like EUMODIC (European Mouse Disease Clinic, www.eumodic.org), which started in 2002, generated and phenotyped 500 and more mutant mouse lines (data available at www.europhenome.org) and are now followed up by the IKMC (International Knockout Mouse Consortium, www.knockoutmouse.org) and IMPC (International Mouse Phenotyping Consortium, www.mousephenotype.org), who will produce and phenotype mutant mouse lines for the rest of the 20,000 plus genes. One of the phenotyping institutions is the German Mouse Clinic (GMC, www.mouseclinic.de) at our research center. Here genetically modified mice are comprehensively phenotyped under standardized conditions in 14 different disease fields (Gailus-Durner et al., 2005; Fuchs et al., 2011, 2012). This phenotyping battery does not yet include a test for stress responsivity. As mutant mice are extremely valuable, and sometimes poor breeders, it is necessary to gain as much information as possible out of one cohort of animals. For this kind of large-scale screening it is important that the collection of data is mostly non-invasive and that the results of the tests applied are reproducible. The gold standard for measuring HPA-axis activity is analyzing CORT levels in blood, the sampling procedure of which is invasive to the animal. Although many protocols have been described in the literature to measure stress responses in rodents, none has been proven to reliably and non-invasively detect stress responsivity phenotypes in mutant mouse lines on a C57BL/6 genetic background.

Here we demonstrate that stress-responsivity can be measured non-invasively by stressing the animal through restraint and simply observing behavior in an Open Field (OF). We also describe the determinants for reproducible results. Restraint was chosen because it is a psychophysical stressor that does not physically harm the animal, and because it is one of the most commonly used stressors (Galvin et al., 1994; Buynitsky and Mostofsky, 2009). The protocol we developed is easy and inexpensive to apply and provides first and foremost reproducible results, which makes it suitable for large-scale screening. It was established for C57BL/6 mice, as mouse mutants generated by the IKMC are on this genetic background, but as we show it also works in BALB/cAnNCrl and C3H/HeNCrl mice, but not in 129S2/SvPasNCrl mice. Different stress durations, i.e., 15 min and 2 h of restraint, can be used to discriminate hyper- and hypo-responsive mutant mouse lines from their respective control lines.

## Materials and methods

### Animals

Wildtype mice were obtained from Charles River (Sulzfeld, Germany) or bred in-house. If not mentioned differently experiments were conducted with male C57BL/6J mice. BALB/cAnNCrl, C3H/HeNCrl and 129S2/SvPasNCrl are referred to as BALB/c, C3H and 129S hereafter. Mutant mouse lines (see below) came from the Max Planck Institute (MPI) of Psychiatry (Munich, Germany). After arrival the animals were left undisturbed for at least 1 week. Animals were group-housed (if not mentioned otherwise) in IVCs (individually ventilated cages) (Techniplast, Buguggiate, Italy) under a 12 h light/dark cycle (lights on at 7:00 AM) with *ad libitum* access to food (Altromin 1314) and water. Room temperature was kept constant at 22°C ± 1°C with a humidity of ~50%. Experiments with wildtype animals began at the age of 8–10 weeks (if not mentioned otherwise), with an age range of 1 week within a cohort.

All experiments were approved by the government of Upper Bavaria, Germany.

### Mutant mouse lines

#### CRH over-expressing mouse line

Mice conditionally over-expressing CRH in the central nervous system (CRH-COE^CNS^) were generated as previously described (for detailed description see Lu et al., 2008). Briefly, conditionally over-expressing (COE) and respective control mice (Ctrl) were obtained by breeding male *R*26^*flopCRH/flopCrh*^ Nes-Cre (**fl**oxed st**op**: flop) mice to female *R*26^*flopCRH/flopCrh*^ mice. CRH over-expression is driven by the *ROSA26* promoter and spatially restricted to the CNS by the nestin (Nes) promoter driving Cre expression. Mice were generated on a mixed 129S2/SvPas × C57BL/6J background and backcrossed to C57BL/6N for five generations. Genotyping primers and protocols are available upon request. Two cohorts of male mice were used. The first cohort was subjected twice to a 15 min stress duration at the age of 25–33 weeks at first stress exposure, with an inter trial interval of 2 weeks. The second cohort also underwent the 15 min restraint stress duration, after a first exposure to the OF without stress 1 week earlier (age at first stress: 16–17 weeks).

#### CRHR1 knockout mouse line

The corticotropin-releasing hormone receptor type 1 knockout (CRHR1-KO) mice were generated as previously described (for detailed description see Timpl et al., 1998). CRHR1-KO and respective wild-type (WT) littermates were obtained by breeding heterozygous male KO mice with heterozygous female KO mice. Mice were kept on a mixed 129P2/OlaHsd × C57BL/6J × CD1 background. Genotyping primers and protocols are available upon request.

Two cohorts of male animals were used. The first cohort was subjected to a 15 min restraint stress at the age of 19–24 weeks and the second cohort was subjected to a 2 h restraint stress at the age of 16–21 weeks.

### Stress protocol

**Figure 1:**
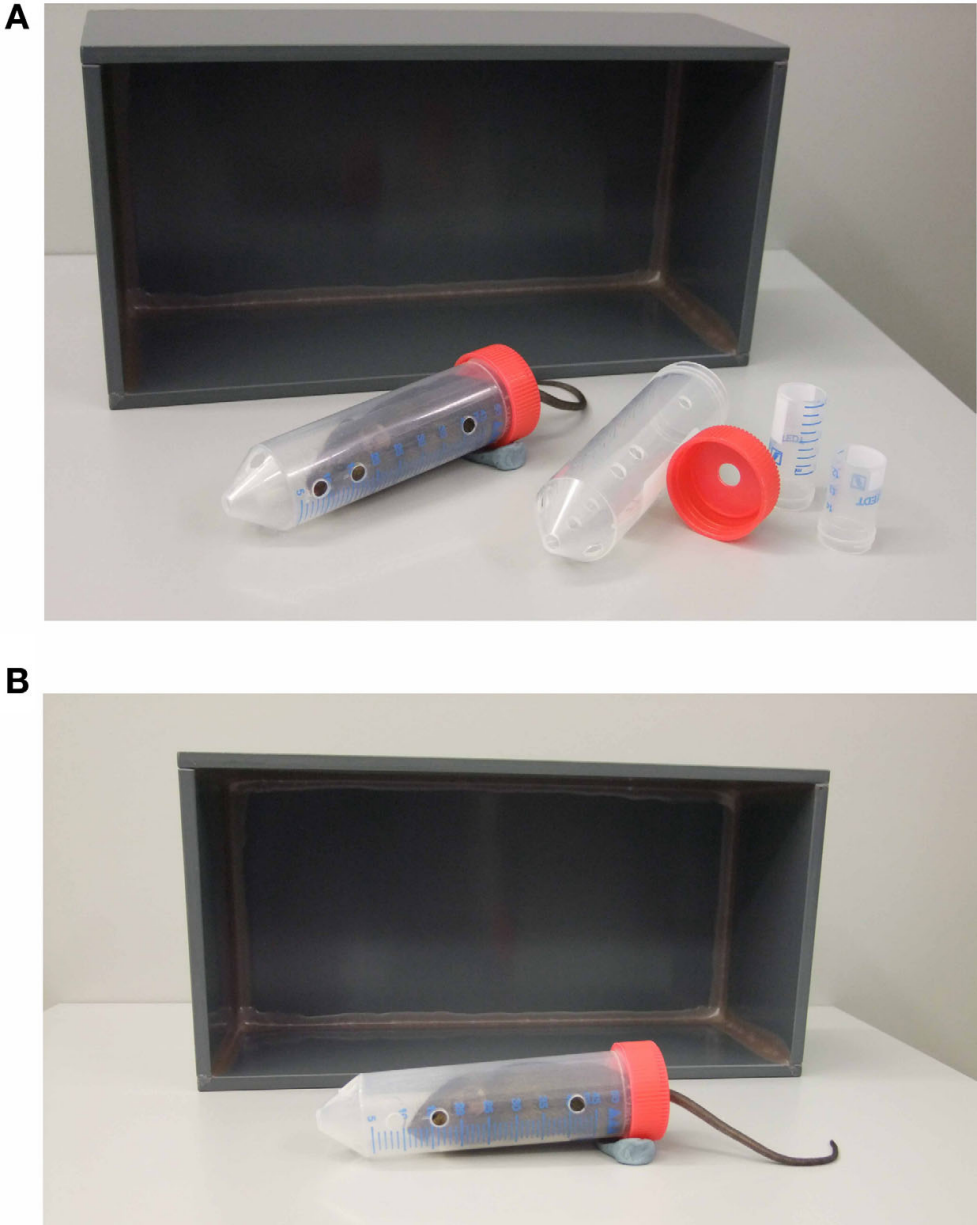
**Acute restraint stress equipment and set up**. The necessary equipment used for the restraint stress is depicted in **(A)**. It consists of a 50 ml restraint tube and cap with holes for breathing and the tail respectively, middle tubes of 4 and 3cm length and a box. The position of the animal inside the restraint tube under the box is depicted in **(B)**. Modeling clay is used to prevent the tube from rolling.

Mice were transferred to the behavioral testing room at least 30 min before the first test to acclimatize. Mice were assigned to one of two groups: either the control group or the stress group. Generally animals of the stress group were restrained in well-ventilated 50 ml tubes (plus 3 or 4 cm long middle tubes, which were slipped over the tail to restrict movement even more (Kim and Han, 2006) and left undisturbed under an opaque box (25 × 12 × 8.5 cm; see Figure 1) in a separate room from the control group for the duration of the stress (i.e., 15 min, 50 min or 2 h). After the restraint period the mouse was transferred into a clean animal housing cage for a 20 min interval and thereafter went through the first behavioral test. Control animals were taken directly from their housing cage into the behavioral test arena.

For evaluation of the stress duration nine independent wildtype C57BL/6 cohorts were obtained. Cohorts 1 and 2 were exposed to a 15 min stress duration, cohorts 3–5 to 50 min restraint and cohorts 6–9 to the 2 h stress duration. Directly after stress cessation mice received a 20 min interval in a clean housing cage, after which they were placed into the Open Field (OF) Test. Cohorts 2 and 9 were of the C57BL/6N strain, all others from the C57BL/6J strain.

To evaluate the necessity of the 20 min interval between stress and OF testing, the stress protocol was also once applied without it and OF behavior was videotaped for measuring grooming behavior.

### Open field test

To measure stress-induced behavioral differences we used the OF. The OF (ActiMot, TSE, Bad Homburg, Germany) is a square (45.5 × 45.5 × 39.5 cm) arena, illuminated with 200 lux in the center, where the animal is traced by a system depending on infra-red light beam breaks (52 Hz, 28 mm apart). The mouse's center of gravity is calculated depending on the number of interrupted beams. A number of 34 parameters are collected automatically in a 10–20 min trial (Activity settings at >0 cm/s; Rearings: minimum duration 200 ms).

For comparison between the two automated systems (ActiMot and EthoVision system) the animal was placed into the OF (ActiMot) with a monochrome camera above it tracing the mouse by the EthoVision system (Version 3.1.16, Noldus Information Technology, The Netherlands; 12.5 Hz; Activity settings: minimum distance moved: 1 cm).

### Corticosterone experiment

Naive wildtype C57BL/6J males were single-housed. Blood samples from each animal were taken at three different time points: basal (*t* = 0), post stress (*t* = 2.00, 2:20 or 2:40 h) and recovery (*t* = 5.00 h). Animals were divided into 5 groups: Control 1 (*t* = 2.20 h, for comparison before OF); Control 2 (*t* = 2.40 h, for comparison after OF); Stress 1 (post stress sample taken at *t* = 2.20 h); Stress 2 (post stress sample taken at *t* = 2.40 h, after OF); Stress 3 (post stress sample taken at *t* = 2 h, before the interval and OF) (see Figure 4). Stressed animals were restrained for 2 h in darkness, had a 20 min interval, and where indicated, were subjected to the OF. Blood, from tail nicks and after decapitation for the last time point, was collected into Microvettes (Sarstedt, Germany) left to coagulate, centrifuged and the supernatant was collected and stored at −20°C until further processing. Plasma corticosterone (CORT) concentrations were measured by a commercially available radioimmunoassay kit (MP Biomedicals, Irvine, CA, USA) according to the manufacturer's instructions.

### Pharmacological experiment

Metyrapone: Naive male C57BL/6J animals were single-housed upon arrival. Animals were assigned to one of the four different treatment groups: Vehicle-injected control (unstressed) animals, vehicle-injected stressed animals, metyrapone-injected control animals or metyrapone-injected stressed animals. Animals received two i.p. injections at a volume of 7 μl/g body weight. The first injection 12 h prior to stress (Metyrapone: 150 mg/kg body weight, Sigma-Aldrich, St. Luis, USA) and the second injection directly before stress (Metyrapone: 100 mg/kg body weight). Control animals were vehicle-injected in parallel. Metyrapone was dissolved in propyleneglycol (Sigma-Aldrich, Steinheim, Germany) and saline (40:60%). After a stress exposure of 2 h animals of the stress-group were placed into a clean cage for the 20 min interval. Thereafter stressed animals and control animals were tested in parallel in the OF. Metyrapone is an 11-beta-hydroxylase inhibitor, thus blocking CORT synthesis (Plotsky and Sawchenko, 1987). The metyrapone doses we used have been shown to suppress stress-induced CORT for several hours (Plotsky and Sawchenko, 1987; Tarcic et al., 1998).

RU486: Upon arrival C57BL/6J males were single-housed and divided into four groups: Vehicle-injected controls, vehicle-injected stressed, RU486-injected controls and RU486-injected stressed animals. All animals received an i.p. injection 1 h pre-stress or in case of the controls 3:20 h pre-OF. Stressed groups underwent a 2 h stress duration after which they were put into a clean cage for 20 min and thereafter were placed in the OF. RU486 (Tocris Bioscience, Missouri, USA) was injected at dose of 25 mg/kg body weight in a volume of 3 μl/g body weight. As RU486 was dissolved in DMSO vehicle-injected animals received DMSO alone. RU486, also known as mifepristone, is a potent GR antagonist. The dose was chosen based on previous studies (Friedman et al., 1997; Flint and Tinkle, 2001; Wamsteeker Cusulin et al., 2013).

### Statistical analysis

For the statistical analysis the program SigmaPlot (Version 11.0; Systat Software, Inc, Chicago, USA) was used. In cohorts with two groups a Student's-*t*-test was applied, in cohorts with more than two groups a One-Way ANOVA or a Two-Way ANOVA were performed where appropriate. In case that normality or equal variance test failed, a Mann-Whitney Rank Sum Test or a One Way ANOVA on Ranks was applied respectively. A *p*-value ≤ 0.05 was considered statistically significant and a value between 0.1 > *p* > 0.05 was considered a trend. Data is shown as mean ± SE of mean (s.e.m.).

## Results

**Figure 2:**
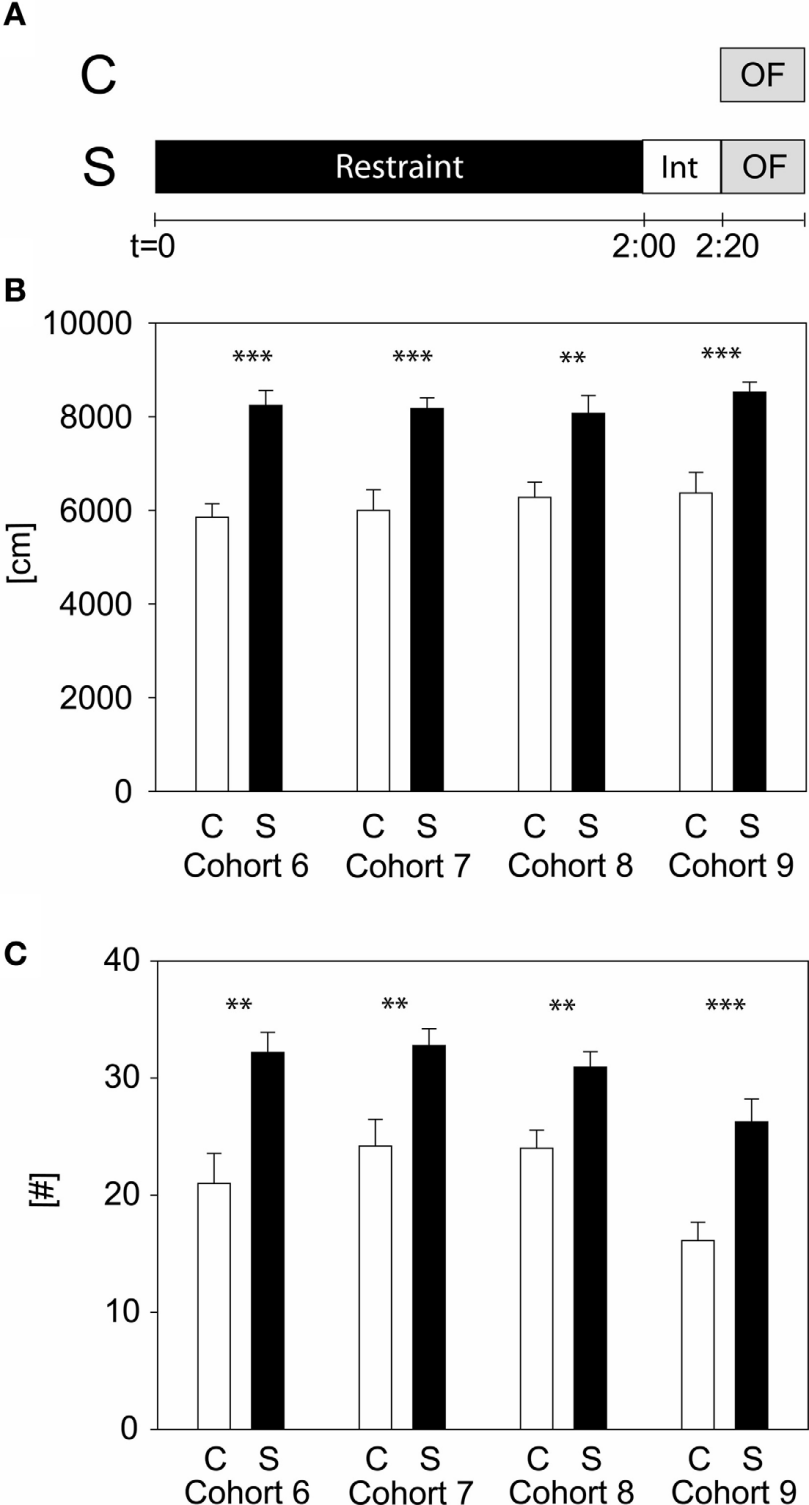
**Two hours acute stress in C57BL/6 males**. Different cohorts of wildtype C57BL/6 strains were tested with the 2 h acute stress protocol. Scheme of experimental design **(A)**. Depicted are the distance traveled **(B)** and the number of rearings **(C)** in the first 5 min of the OF. Control groups, C, in white and stress groups, S, in black bars. Note that cohort 9 is of the C57BL/6N strain, whereas all the other cohorts are of the C57BL/6J strain. Int, interval; Error-bars shown as s.e.m.; significances: ^**^-*p* ≤ 0.01; ^***^-*p* ≤ 0.001 vs. C; *n* = 11–16 per group.

To evaluate which stress duration would lead to reproducible results, we applied several different durations of restraint stress: 15, 50 min, and 2 h to independent cohorts of naïve animals (cohorts 1–9). Thirty-four parameters, including time spent and distance traveled in the center, were analyzed for the OF. Only the 2 h stress duration showed reproducible stress-induced differences in behavior, namely during the first 5 min of the OF in distance traveled and number of rearings (see Table 1 and Supplementary Material). These behavioral changes were reproduced in all cohorts of both C57BL/6J (cohort 6, 7, and 8) and C57BL/6N (cohort 9) (see Figure 2), although methods differed slightly. In cohort 6, 8, and 9 animals were restrained with a 3 cm long middle tube and under a box, whereas in cohort 7 animals were restrained with a 4 cm long middle tube and at 160 lux. As can be seen from Figure 2, these small methodological differences still lead to the same robust result in both the distance traveled and the number of rearings (distance traveled: cohort 6: *t*_22_ = −5.58, *p* ≤ 0.001; cohort 7: *t*_22_ = -4.61, *p* ≤ 0.001; cohort 8: *t*_22_ = −3.61, *p* ≤ 0.01; cohort 9: *U* = 19.0, *p* ≤ 0.001; number of rearings: cohort 6: *t*_22_ = −3.6, *p* ≤ 0.01; cohort 7: *t*_22_ = −3.29, *p* ≤ 0.01; cohort 8: *t*_22_ = −3.41, *p* ≤ 0.01; cohort 9: *t*_22_ = −4.08, *p* ≤ 0.001).

**Table 1:** **First 5 min of the OF with different stress durations and cohorts**.

**Stress duration**	**15 min**		**50 min**			**2 h**			
**Cohort**	**1**	**2**	**3**	**4**	**5**	**6**	**7**	**8**	**9**
Strain: C57BL/6…	J	N	J	J	J	J	J	J	N
Sample size (C/S)	8/8	12/12	12/12	12/12	8/12	12/12	11/13	12/12	16/12
Distance traveled	ns	^*^↑	T↑	^***^↑	^***^↑	^***^↑	^***^↑	^**^↑	^***^↑
Number of rearings	ns	ns	ns	^**^↑	^**^↑	^**^↑	^**^↑	^**^↑	^***^↑

*The interval duration was always 20 min; all animals tested were males. Arrows indicate the direction of the effect compared to unstressed controls*.

*** p ≤ 0.001;

** p ≤ 0.01;

* *p ≤ 0.05; T-p < 0.1; ns, not significant; C, control group, S, stressed group*.

Observations of the animals during the 20 min interval after the 2 h stress duration showed that they spent some time grooming. This was due to the fact that some of the animals urinated in the restraint tubes and thus were wet when placed into the clean cage for the time of the interval. After the 20 min interval animals were dry again. Therefore we investigated if the 20 min interval between stress and the OF test was necessary for the reliability of the increase in distance traveled and number of rearings during the first 5 min of the OF test. To this end we performed one experiment restraining animals for 2 h and directly thereafter placing them into the OF arena. Neglecting the interval lead to a significant, nearly ten fold increase in grooming behavior in stressed animals (data not shown; time spent grooming in the first 5 min of the OF: *U* = 0.00, Con vs. Stress: *p* ≤ 0.001). The effect of stress in distance traveled was still significant although less strong (*t*_21_ = −2.44, *p* ≤ 0.05), but a stress-induced difference in number of rearings was not observable (Con: mean: 10.4, s.e.m.: 2.3; Stress group: mean: 10.3, s.e.m.: 1.4). This result demonstrated that a 20 min interval after stress exposure and before further behavioral testing is necessary to not confound other behavioral read-outs by enhanced grooming.

**Figure 3:**
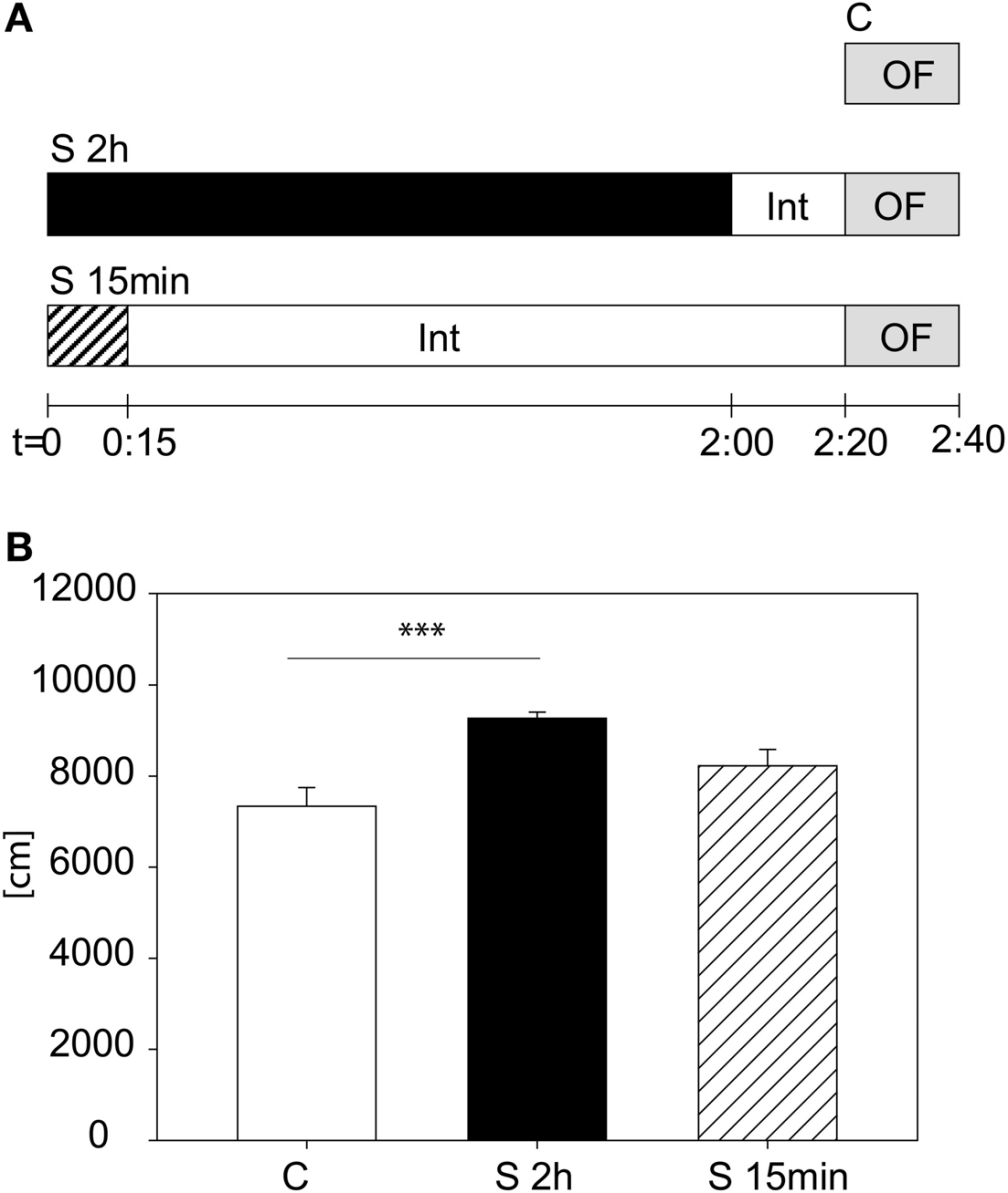
**Stress duration and interval between beginning of stress and beginning of the Open Field Test**. Scheme of experimental design **(A)**. Distance traveled in the first 5 min of the Open Field Test **(B)**. A significant difference was observed between the 2 h stressed and control animals. Control group, C, in white, 2 h stress group, S 2 h, in black and 15 min stress group, S 15 min, in striped bars. Int, interval; Error-bars are shown as s.e.m.; Significances: ^***^p ≤ 0.001; *n* = 8.

We tested another cohort to evaluate whether it is the stress duration or the time-lag between the beginning of the stress and the start of the OF that are relevant for the behavioral changes in the first 5 min of the OF. We assigned animals to three different groups (see Figure 3A): A control group (C), a stressed group (S 2 h), with the 2 h restraint plus the 20 min interval and a third group (S 15 min), being stressed for 15 min with a subsequent interval of 2:05 h. In Figure 3B the result for the distance traveled in the first 5 min is depicted. The One-Way-ANOVA revealed a significant difference between the groups [*F*_(2, 23)_ = 8.83, *p* ≤ 0.01; post hoc Holm-Sidak: C vs. S 2 h: *t* = 4.2, *p* ≤ 0.001; C vs. S 15 min: n.s.], suggesting that although the 15 min stress period also led to a tendential increase in activity during the first 5 min in the Open Field 2 h later, the 2 h stress period yields this increased activity more reliably.

**Figure 4:**
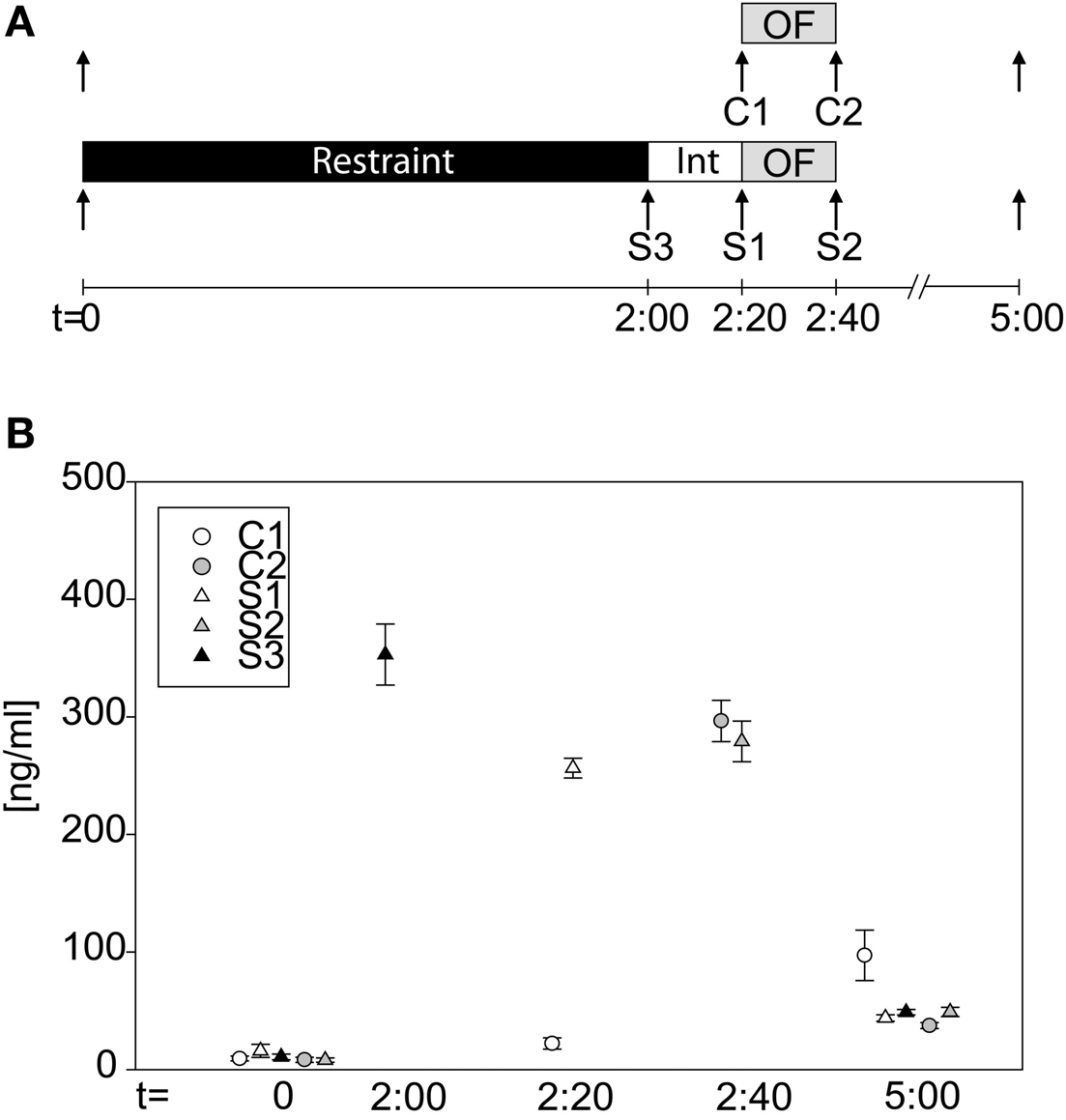
**Corticosterone profile during the acute stress test. (A)** Scheme of the experimental design. Arrows indicate time point of blood withdrawal. C1 and C2 are control groups. Stressed groups, S1–S3, were exposed to a 2 h restraint stress period (black bar) and a 20 min interval (white bar) before they were placed into the OF. **(B)** CORT levels at the different time points of the different groups. At basal (*t* = 0 h) no differences in CORT levels can be seen. Directly after stress (S3), after the interval (S1) and after the OF (S2) CORT levels of the stressed animals are high. CORT levels of the control group, C1, are low at *t* = 2.20 h, whereas they are increased after the OF (C2). At *t* = 5 h all groups have low circulating CORT levels again. Int, interval; *n* = 11–12 per group.

To actually verify that our 2 h restraint stress leads to activation of the HPA-axis, we took blood samples at different time points (see Figure 4A). Groups did not differ in baseline levels at *t* = 0 (One-Way-ANOVA on ranks: n.s.). After the 2 h restraint period stressed animals clearly showed an increase in CORT levels, which remained high even after the 20 min interval (S3_basal_ vs S3_*t* = 2:00_: *U* = 0.0, *p* ≤ 0.001; C1_*t* = 2:20_ vs. S1_*t* = 2:20_: *t* = −25.42, *p* ≤ 0.001). The OF exposure itself can activate the HPA-axis, as can be seen at time point 2:40 h, after the OF, when both stressed and control groups showed high CORT levels (C2_*t* = 2:40_ vs. S2_*t* = 2:40_: n.s.). Three hours after cessation of stress or, as in case of the control mice, 5 h after first blood withdrawal, all groups showed low levels of CORT again (One-Way-ANOVA on ranks: *H* = 13.05, *p* ≤ 0.05). The C1 group showed higher values compared to the other four groups, which might be due to a missing stressor (as all other groups were subjected to restraint or in case of the C2 group to a 20 min OF), thus causing a slight increase in CORT secretion at the third blood withdrawal.

**Figure 5:**
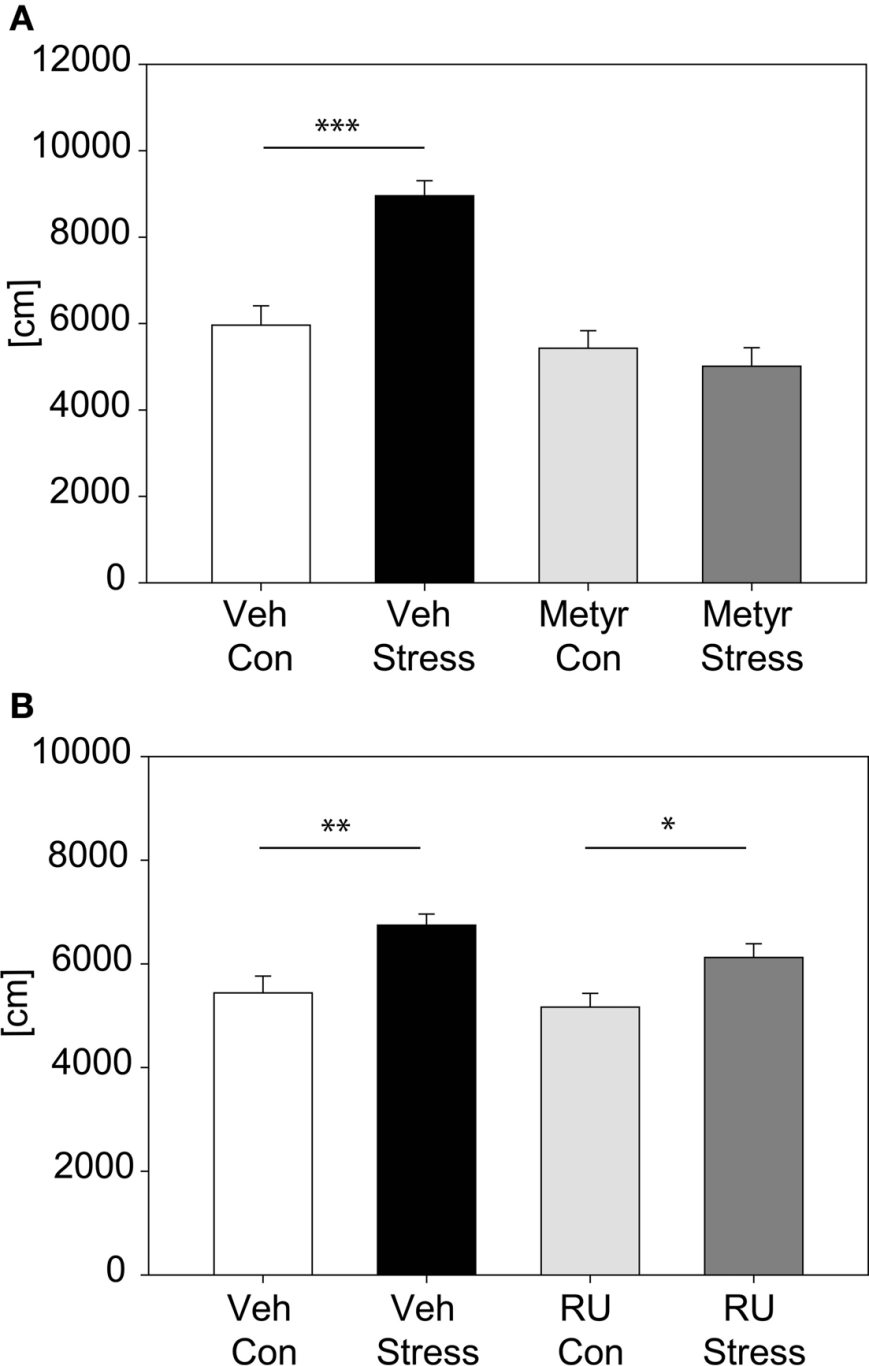
**Pharmacological analysis of corticosterone feedback**. Graph **(A)** shows the distance traveled in the first 5 min of the OF with animals treated with Metyrapone or vehicle. Statistics revealed a significant interaction and *post hoc* tests demonstrated that only in the vehicle-treated animals the expected increase after stress occurred. Graph **(B)** depicts the distance traveled in the first 5 min of the OF with animals treated with either RU486 or vehicle. Vehicle-treated as well as RU486-treated animals show an increase in activity after 2 h of stress. Error-bars shown as s.e.m.; Significances: ^*^p ≤ 0.05; ^**^p ≤ 0.01; ^***^p ≤ 0.001; *n* = 11–15 per group; Veh, vehicle; Metyr, Metyrapone; RU-RU486; Con, control group; Stress, stressed group.

The involvement of CORT in the behavioral response to our stress exposure was evaluated by applying a pharmacological approach; we inhibited CORT synthesis by metyrapone (see Figure 5A). Results show that animals injected with metyrapone did not increase their activity in response to a 2 h acute stress [distance traveled: interaction: *F*_(1, 46)_ = 17.22, *p* ≤ 0.001; *post hoc*: Holm-Sidak: Vehicle: Con vs. Stress: *t* = 5.21, *p* ≤ 0.001; Metyrapone: Con vs. Stress: n.s.]. In the second pharmacological experiment (Figure 5B) we blocked the GR by injecting RU486. There was no significant interaction between stress and treatment, thus there was no difference between vehicle- and RU486-injected animals in response to stress, only a general stress effect was observed [stress effect: *F*_(1, 55)_ = 17.05, *p* ≤ 0.001].

**Figure 6:**
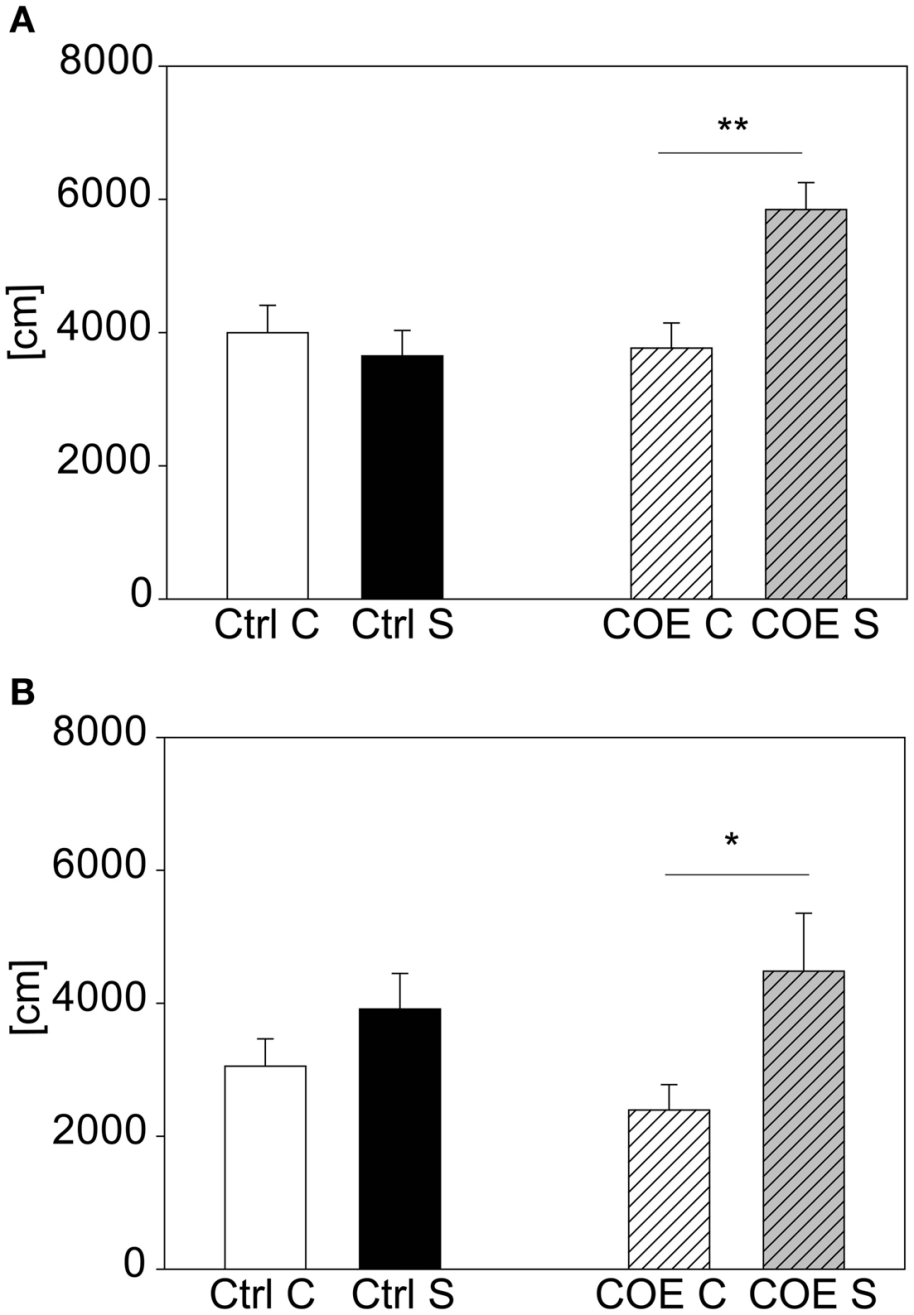
**CRH-COE^CNS^ line**. Graphs depict the distance traveled during the first 5 min in the OF. Both control (Ctrl) and CRH over-expressing (COE) littermates of the more stress-reactive CRH-COE^CNS^ line were exposed to a 15 min restraint stress period. At the second exposure there was a significant interaction between genotype and stress effect. Control littermates did not respond to the stress with increased activity, whereas the CRH over-expressing mice did **(A)**. The second cohort confirmed the result of higher stress-responsivity in the CRH over-expressing mice **(B)**. Unstressed groups, C, in white or white striped and stress groups, S, in black or gray striped bars. Error-bars shown as s.e.m.; Significances: ^**^p ≤ 0.01; ^*^p ≤ 0.05; *n*_(cohort 1)_ = 7–11 per group; *n*_(cohort 2)_ = 5–9 per group.

For validating our acute stress test and to prove the possibility to detect both hyper- and hypo-responsive phenotypes in mutant mouse lines, we selected two different mouse lines with known differences in response to stress. The CRH-COE^CNS^ mouse line, over-expressing CRH, was challenged with 15 min restraint stress, since we reasoned that this shorter stress duration is milder and more suitable to reveal hyper-responsivity to stress than the 2 h stress duration that reliably produces a clear behavioral response in wildtype C57BL/6. After the first acute 15 min stress exposure there was a significant interaction between genotype and stress [*F*_(1, 37)_ = 4.22, *p* ≤ 0.05]. *Post hoc* testing revealed no effect in the littermate controls but a trend in the over-expressing mice (*t* = 1.72, *p* = 0.094). The test was repeated a second time 2 weeks later. This time statistical analysis revealed a significant interaction between genotype and stress [*F*_(1, 37)_ = 8.04, *p* ≤ 0.01], which was driven by the conditional over-expressing mice (*post hoc*: Ctrl: Con vs. Stress: n.s.; COE: Con vs. Stress: *t* = 3.4, *p* ≤ 0.01; see Figure 6A). The second cohort of animals confirmed the former finding, in that the CRH over-expressing mice responded to the stressor and the controls did not [stress effect: *F*_(1, 27)_ = 7.69, *p* ≤ 0.05; *post hoc*: Ctrl: Con vs. Stress: n.s.; COE: Con vs. Stress: *t* = 2.59, *p* ≤ 0.05; see Figure 6B].

**Figure 7:**
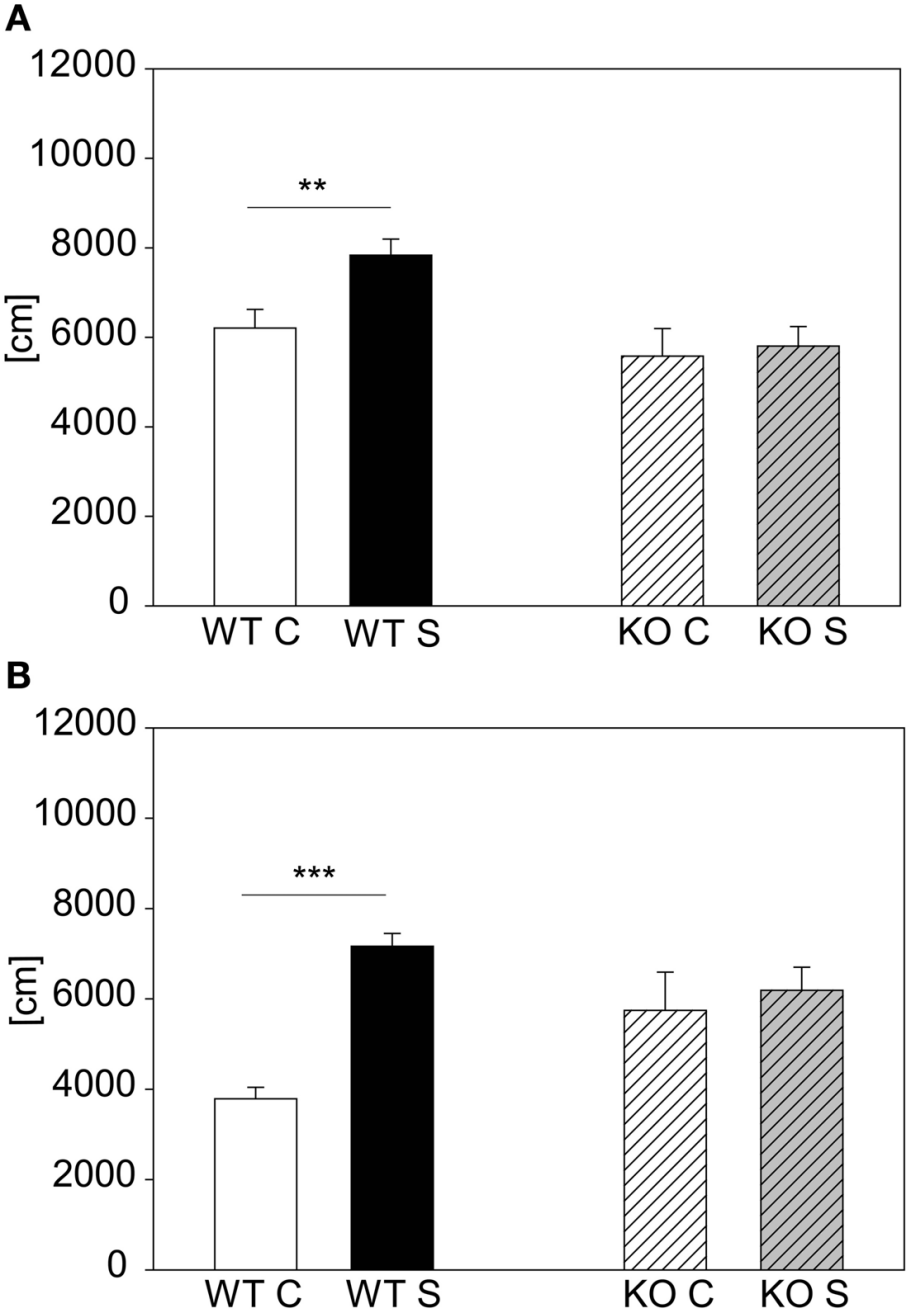
**CRHR1-KO line**. Graphs depict the distance traveled during the first 5 min in the OF. For the animals from the more stress-resistant line, the CRHR1-KO line, the 15 min restraint stress period only had an effect in the wildtypes (WT) **(A)**. This effect of reduced responsivity to stress in mutants (KO) was even clearer in a second cohort of animals with a 2 h exposure to restraint **(B)**. Wildtypes showed an increased responsivity, whereas the stressed mutants did not show any difference compared to their unstressed controls. Control groups, C, in white or white striped and stress groups, S, in black or gray striped bars. Error-bars shown as s.e.m.; Significances: ^**^p ≤ 0.01; ^***^p ≤ 0.001; *n* = 7–13 per group in the first cohort; *n* = 4–6 in the second cohort.

The other mutant mouse line we chose for validation was the less stress-reactive CRHR1-KO mouse line. In contrast to the CRH-COE^CNS^ mouse line the CRHR1-KO mutants did not respond to the 15 min stress duration, although the wildtype littermates did [first cohort: genotype effect: *F*_(1, 43)_ = 8.5, *p* ≤ 0.01; stress effect: *F*_(1, 43)_ = 4.12, *p* ≤ 0.05; post hoc: WT: Con vs. Stress: *t* = 2.88, *p* ≤ 0.01; KO: Con vs. Stress: n.s.; see Figure 7A). The second cohort of CRHR1-KOs strengthened the results from the first cohort by showing that even after 2 h of stress the mutants did not react to stress [interaction: *F*_(1, 17)_ = 6.73, *p* ≤ 0.05; *post hoc*: WT: Con vs. Stress: *t* = 4.22, *p* ≤ 0.001; KO: Con vs. Stress: n.s.; see Figure 6B]. Taken the results from both mutant mouse lines together, we could show that we can detect hyper-responsive mice with the 15 min stress duration and hypo-responsiveness with the 2 h restraint duration.

As there are many methodological ways to analyze locomotor behavior, we explored the differences between two systems, namely the ActiMot and the EthoVision system. The differences in locomotor activity between the control and the stressed group can be detected by both systems, but there is a clear system-group-interaction, as revealed by the Two-Way ANOVA [F_(1, 35)_ = 5.01, *p* ≤ 0.05, *post hoc* Holm-Sidak: ActiMot: C vs. S: *t* = 5.25, *p* ≤ 0.001, EthoVision: C vs. S: *t* = 2.09, *p* ≤ 0.05]. Looking at percent time spent in the center of the OF, no significant differences between systems and groups could be detected, which shows that differences between these two systems only exist in activity measurements, such as the distance.

**Figure 8:**
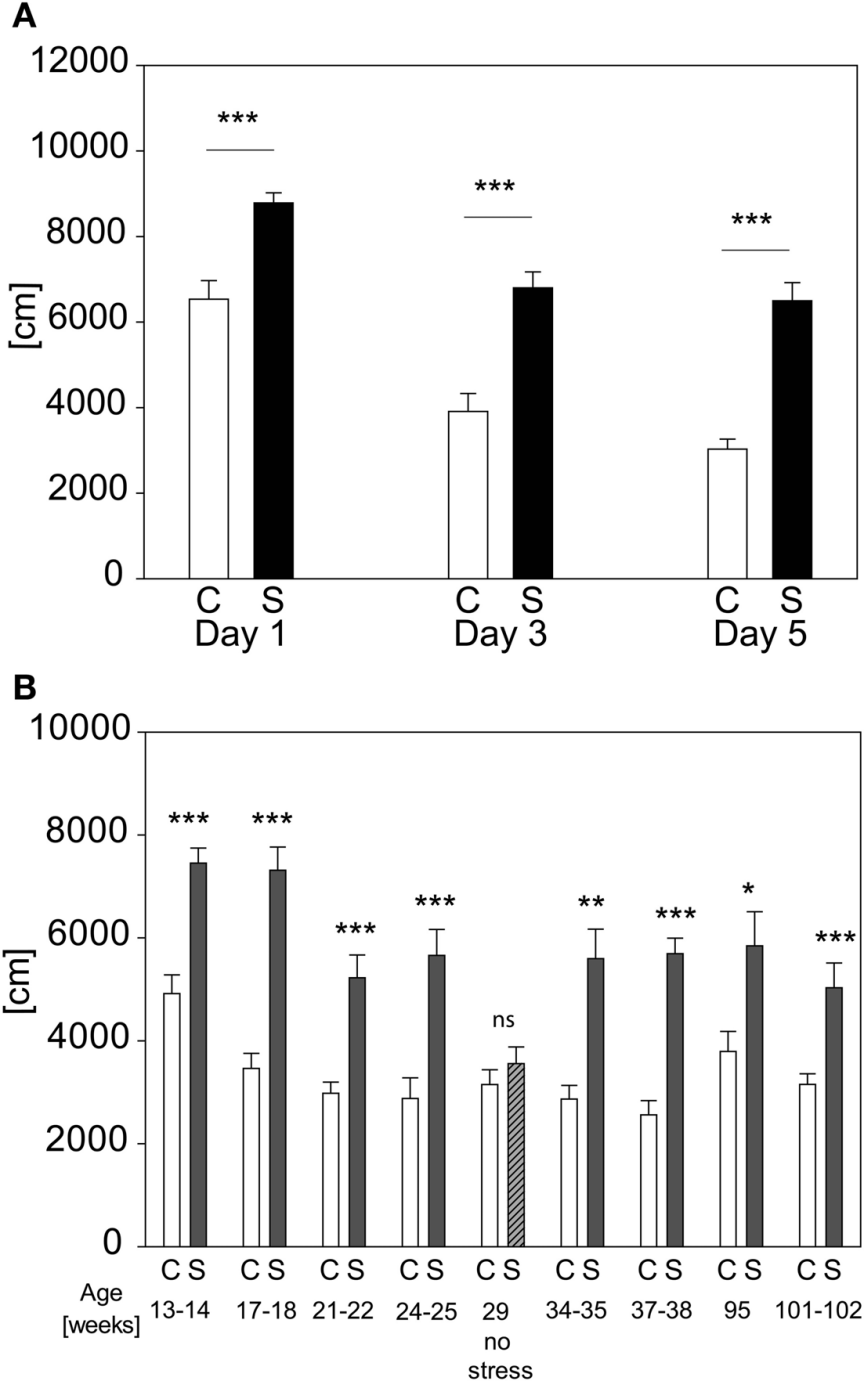
**Repeated exposure to acute stress**. When re-testing the same animals every second day the increased activity in distance traveled **(A)** in the first 5 min of the OF persists and does not fade-out nor vanish. In graph **(B)** the distance traveled in the first 5 min of the OF after repeated exposure to the acute restraint stress protocol throughout life time is depicted. At the age of 29 weeks the stressed group was tested for development of a conditioned response to the OF, by not stressing them, but placing them into the OF directly from the home cage, as done with controls. The lack of an increase of activity demonstrates that the animals of the stress group did not respond to the environment they were placed into but to the actual stressor, namely restraint. Control groups, C, in white and stress groups, S, in black bars. Error-bars are shown as s.e.m.; Significances: ^*^p ≤ 0.05; ^**^p ≤ 0.01; ^***^p ≤ 0.001 vs. C; *n* = 12 per group; gradual decline in animal numbers in graph **(B)** to *n*_(C)_ = 11 and *n*_(S)_ = 7 at the end of repeated stress exposure.

We evaluated the possibility of re-testing animals with the acute stress test for several times. Retesting every second day for three times leads to stress-induced differences on every day in distance traveled [see Figure 8A; stress-day interaction: *F*_(2, 71)_ = 3.175; *p* ≤ 0.052; stress effect: *F*_(1, 71)_ = 43.962, *p* ≤ 0.001; *post hoc*: Day 1: C vs. S: *t* = 4.37, *p* ≤ 0.001, Day 3: C vs. S: *t* = 5.61, *p* ≤ 0.001; Day 5: C vs. S: *t* = 6.73, *p* ≤ 0.001]. Another cohort was retested throughout lifetime (see Figure 8B). It can be seen that an adaption to the OF in both groups occurred as illustrated by the reduction of the absolute levels of the behavioral response with repeated exposures, but the differences between groups stayed significant (*t*-tests for the individual time points: Age 13–14 weeks: *t*_22_ = −5.3964, *p* ≤ 0.001; age 17–18 weeks: *t*_21_ = −7.270, *p* ≤ 0.001; age 21–22 weeks: *U* = 11.0, *p* ≤ 0.001; age 24–25 weeks: *t*_21_ = −4.344, *p* ≤ 0.001; age 29 weeks (no stress): n.s.; age 34–35 weeks: *U* = 11.0, *p* ≤ 0.001; age 37–38 weeks: *t*_20_ = −7.635, *p* ≤ 0.001; age 95 weeks: *t*_16_ = −2.861, *p* ≤ 0.05; age 101–102 weeks: *t*_16_ = −4.104, *p* ≤ 0.001). In order to assess the possibility that conditioning occurred in the stressed group, we analyzed the behavior of all animals without any prior stressor at the fifth exposure (compare age 29 weeks “no stress”). As depicted in Figure 8B no differences can be seen between groups at the time point “no stress,” indicating that the behavioral response of the stressed group had not become conditioned to the environment of the OF arena.

**Figure 9:**
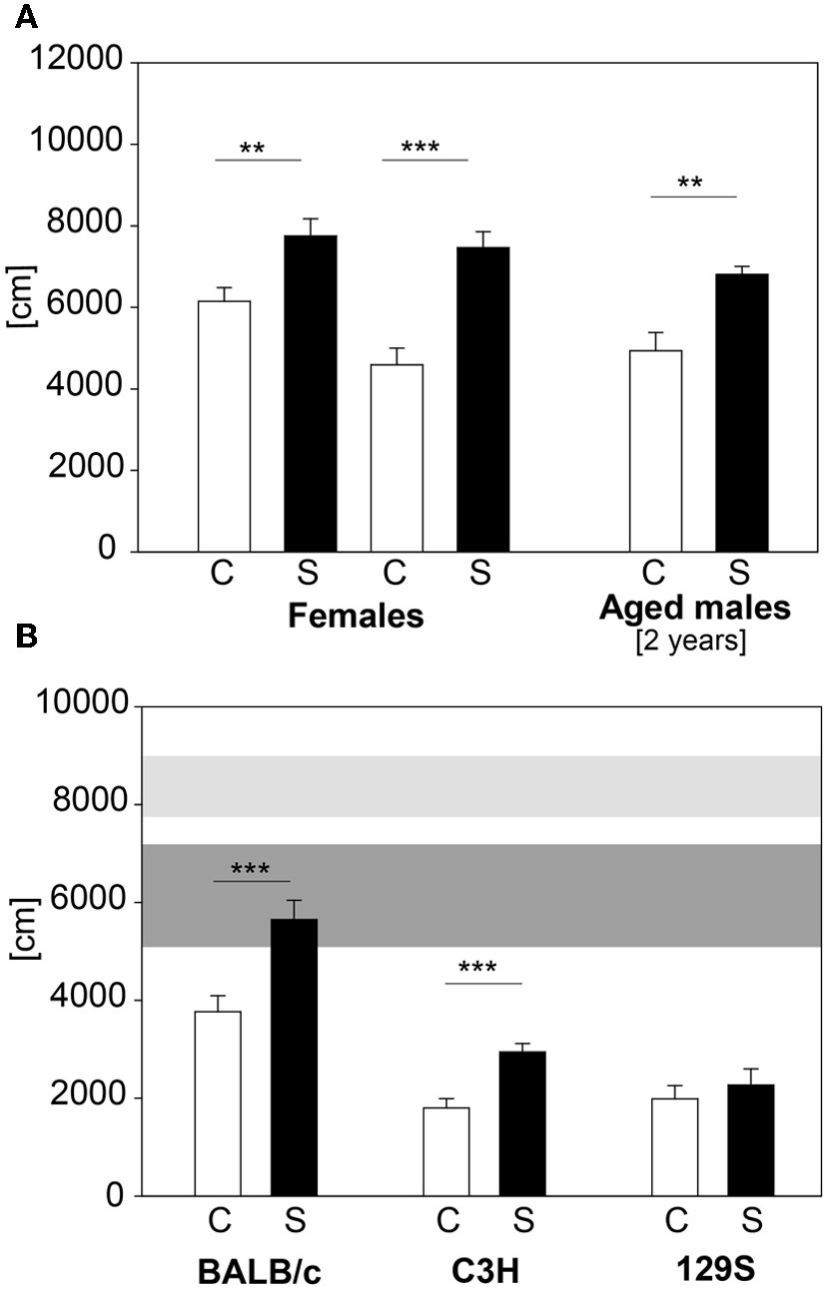
**Female and 2 year old male C57BL/6 and different inbred strains. (A)** Female C57BL/6J mice were tested with the 2 h restraint stress test. In both exposures females showed the expected increase in distance traveled. Aged C57BL/6J males also showed the increase in distance traveled. **(B)** BALB/c, C3H and 129S wildtype males were also tested with the 2 h acute stress test. All of these strains demonstrated a lower locomotor activity compared to the C57BL/6 strain (horizontal shades). Both BALB/c and C3H showed increased activity after stress. Only the 129S strain did not show the behavioral response after stress exposure. Control groups, C, in white and stress groups, S, in black bars. Horizontal shades represent the 25–75% percentile of four male C57BL/6 cohorts (*n*: Con = 53, Stress = 49). Dark gray shade: C57BL/6 control groups; light gray shade: C57BL/6 stress groups; Significances: ^**^p ≤ 0.01; ^***^p ≤ 0.001; *n* = 11–13 per group.

In large-scale screening not only male C57BL/6 mice are analyzed, but also females and mutant mice with varying genetic background. Therefore we applied our established protocol to female C57BL/6 animals, 2 year old C57BL/6 males and males of the BALB/c, 129S and C3H strains. C57BL/6 females showed increased distance traveled in the first 5 min of the OF, but only a trend in number of rearings during this time (distance traveled: *t*_21_ = −3.0, *p* ≤ 0.01; number of rearings: *t*_22_ = −1.84, *p* = 0.08). We retested these females when their body weight reached approximately the same values as 9 week old males. Females showed both an increase in distance traveled (see Figure 9A) and in number of rearings in the first 5 min in the OF test at the second exposure (distance traveled: *t*_21_ = −5.05, *p* ≤ 0.001; number of rearings: *t*_21_ = −2.5, *p* ≤ 0.05; data for number of rearings not shown). The 2-year old male C57BL/6 cohort showed the expected differences (see Figure 9A) in distance traveled (*U* = 9.0, *p* ≤ 0.01). The different wildtype strains tested showed an overall lower locomotor activity than the C57BL/6 males (see Figure 9B). Both BALB/c and C3H demonstrated a stress-induced increase in distance traveled in the first 5 min of the OF (BALB/c: C vs. S: *t*_22_ = −3.68, *p* ≤ 0.001; C3H: C vs. S: *t*_22_ = −4.48, *p* ≤ 0.001), whereas the 129S strain did not respond to the 2 h stress duration with an increase in distance traveled.

## Discussion

Our results demonstrate that the described protocol can be reliably used for non-invasively testing stress-responsivity in mice. The suppression of stress-induced hyperlocomotion by the CORT synthesis inhibitor metyrapone suggests that CORT secretion is necessary for this behavioral response. These acute effects of increased CORT levels, either through stressors or by injections of CORT, on behavior have been described in previous reports (Armario et al., 1990; Sandi et al., 1996; Haller et al., 1998). It also seems clear, that not only the intensity of a stressor plays a role in the behavioral outcome but also the test applied and the timing, a conclusion in line with a review of the abundance of literature on stress.

Restraint is one of the most commonly used stressors in mice and rats to elicit a CORT response. A high variability between the different methods exists. This is not only true for the duration of the restraint, but also for the method of restraint (such as restraint in tubes or with wire mesh, as well as by taping the limbs to a surface; for review see Galvin et al., 1994; Buynitsky and Mostofsky, 2009). These conditions make it differently severe for the exposed animal, and this might not only depend on the method but also on the animals used. For one there are species-specific differences between rats and mice (Armario and Castellanos, 1984; Griebel et al., 1997; Van Pett et al., 2000; Bain et al., 2004), as well as strain-specific differences both in rats and mice (Brinks et al., 2007; Nosek et al., 2008). Together with differences in measurements after stress (such as CORT levels or behavioral tests applied) and at what time point they occur, this makes it extremely difficult to compare results from different studies. Especially the timing of behavioral testing varies greatly, some authors test for emotionality directly after the stress period (Katz et al., 1981; Nosek et al., 2008), others include an interval with varying lengths (for review see Armario et al., 2008). Also the duration of the test itself can influence outcome.

In our acute restraint protocol with the OF as a read-out we can reproducibly see enhanced activation in the first 5 min of the test (i.e., increased locomotion and number of rearings) in the stressed group 20 min after stress cessation, but only then and not at later time points (data not shown). The difference is gone when looking at the total 20 min of the test. The OF is used as a standard behavioral test in our lab, and therefore was applied as a first test for establishing the acute stress protocol. We also tried the Elevated Plus Maze as read-out test, but did not see any consistent changes in any parameter there (data not shown). The 20 min interval between stress and OF is essential for correct interpretation of the results, neglecting it can lead to corruption of the collected data, due to enhanced grooming of the stressed animals after being released from the restrainers. The stress-induced behavioral effects depend on the duration of the stress and not on the time-lag between the onset of stress and the start of OF testing (compare Figure 3). By varying the stress duration we can detect differences in stress-hyper-and hypo-responsivity, as shown for the CRH over-expressing, hyper-reactive CRH-COE^CNS^ line with 15 min of restraint stress and for the hypo-reactive CRHR1-KO line with the 2 h stress protocol.

Measuring CORT levels at different time points have shown that 2 h restraint leads to an increase in CORT levels, which is in line with previous studies (Flint and Tinkle, 2001; Kim and Han, 2006; Bowers et al., 2008). Interestingly the OF itself is stressful for the animal, as also the control group (see Figure 4, group C2) showed increased CORT levels after OF. This effect has also been observed in previous studies (Briski, 1996; Thoeringer et al., 2007; Steward et al., 2008). CORT is responsible for the behavioral manifestation of the stress-response, as shown by the lack of stress-induced increases in activity after blocking CORT production by metyrapone-injections. This experiment suggests that CORT synthesis in response to stress and its rise are a prerequisite for the disclosure of locomotor activity, but as CORT levels and locomotor activity do not correlate in our experiment other factors might influence the magnitude of activity. One should mention that the correlation was made between the distance traveled in the first 5 min of the OF and CORT levels at the end of the 20 min OF. Thus it is not clear if the correlation between those parameters does not exist or has been masked by the increase of CORT during the OF exposure. Further investigations are needed to clarify this point. The stress-induced behavioral response is not mediated via the GR, as demonstrated by the lack of effect of the blockade of this receptor by RU486. It further has to be validated to what extent the MR contributes to the stress-induced behavioral effects. As the behavioral effect appears shortly after stress, we can speculate that the CORT-signal is mediated via a non-genomic mode, possibly through membrane-bound MRs. This has yet to be confirmed, but other studies hint on its relevance. For example Sandi et al. (1996) showed that a single CORT-injection leads to an increased locomotor activity, shortly after injection, which could not be blocked by antagonists of the intracellular MR and GR nor by cycloheximide (a protein synthesis inhibitor), suggesting a non-genomic effect.

Differences in the applied behavior detection systems, here between the ActiMot and EthoVision system, appear to be only present in parameters of activity, like forward locomotion. This discrepancy must be due to the different measuring methods for the activity of an animal. The video-based EthoVision system traces the movement of the mouse by using the mouse's center of gravity, tracking mostly the forward locomotion, and possibly neglecting smaller movements. ActiMot system, which relies on infra-red beam breaks caused by the mouse, traces the activity. Here even smaller movements, like the nose poking back and forth, thereby interrupting light-beams, might cause higher absolute values. Another factor adding to the differences between the systems could be the temporal resolution. Our ActiMot system runs on 52 Hz, whereas the EthoVision system runs with 12.5 Hz. Looking at other parameters measured with both systems, like the time spent in the center, did not reveal any differences, confirming that activity-related measures are the only ones that are affected by the different methods. Nonetheless, the difference between the control and the stressed group is evident in both systems. For application in the acute stress test one would preferentially work with the ActiMot system, since it appears to be more sensitive.

Another essential setting is the individual behavioral testing in the OF in small chambers (1 × 1m). We found that testing in lab rooms, where OF apparatuses are placed next to each other only separated by blinds, did not lead to the expected increase of activity in response to stress (data not shown). This could be due to different influences on the animals, such as the auditory and olfactory cues of other mice being tested in parallel, and a more stimulating environment outside the OF due to shelves and other objects, which are possibly being perceived by the test mouse. Taken together the type of behavioral test (see above) as well as its set-up is essential for the detection of stress-induced increased activity.

Retesting the animals did not lead to behavioral habituation to the stressor as one might expect from biochemical research, where repeated stressing causes a reduction in the HPA-axis response, e.g., ACTH, corticosterone and c-fos expression (Girotti et al., 2006), although there is a habituation to the OF. It even did not seem to matter if the animals were repeatedly stressed 8 times over the course of approx. 2 years or 3 times (i.e., every 2nd day) over the course of 5 days (Figure 8). Still the differences between the control and stressed group can be seen. We could also demonstrate that the stressed animals are not conditioned to the OF itself, because at the age of 29 weeks none of the two groups were stressed and yet no differences between groups could be observed (see Figure 8B at 29 weeks of age).

We also tested female and 2 year old C57BL/6 mice. Both cohorts pointed out the necessity of adjusting the size of the tube to the mouse's body weight, as already suggested by Johnson et al. (2000). Therefore we use middle tubes of varying length for smaller animals, which are slipped over the animal's tail inside the restraint tube to restrict movement even more (compare Figure 1A). In case of larger animals, i.e., old and/or obese mice, larger animal holders are used to restrain the animal instead of 50 ml tubes.

Three different inbred mouse strains were tested in our 2 h stress responsivity test. In general we see that BALB/c, C3H and 129S mice are less active than the C57BL/6 strain, which is in line with the literature (Lhotellier et al., 1993; Crawley and Paylor, 1997; Mandillo et al., 2008). In both the BALB/c and the blind C3H strains we could observe stress-induced increased distance traveled, but not in the 129S. Many studies have shown various differences, behavioral as well as neuroendocrine, between inbred strains (for review see Jacobson and Cryan, 2007). This includes data for stress responsivity, where it was shown that some strains are more sensitive to an acute stress (e.g., BALB/c) compared to less-sensitive strains (e.g., C57BL/6) (Shanks et al., 1990, 1991; Anisman et al., 1998; Browne et al., 2011). The lack of a stress-induced behavioral effect in the 129S strain might be due to a different stress-coping strategy and different transmitter systems activated during stress (Van Bogaert et al., 2006). Still, this might only be true for this tested 129S line and not for other 129 substrains, as well as for mice of mixed genetic background. For example the CRHR1-KO line applied here, is on a mixed background including 129P2/OlaHsd, and we do get the expected stress-induced differences in activity in the wildtype littermates. In this respect it is advisable to always test wildtype and mutant littermates in parallel, as differences in genetic background can contribute to differences in phenotypes, as nicely shown by Holmes et al. (2003). Although BALB/c and C3H mice show a significant stress-induced increase in distance traveled, there is no such increase in number of rearing (data not shown). Again, this might be due to differences in stress-coping and/or transmitter systems (He and Shippenberg, 2000; Yochum et al., 2010) but also strengthens the concept that rearing and locomotor activity are independent behaviors (Gironi Carnevale et al., 1990; Murphy and Maidment, 1999; Cornish et al., 2001; Pawlak and Schwarting, 2002; Lever et al., 2006). Also in our experiments with the C57BL/6 strain the distance traveled and the number of rearings is not correlated in stressed animals. Interestingly, the absolute values of distance traveled in the 2 h restraint in C57BL/6 seem to be more stable than the values of the number of rearings (see Figure 2), which could hint to different underlying processing systems.

In conclusion, here we describe a reliable and robust acute stress test, with which genetically modified mice can be non-invasively tested for their stress-responsivity as an indicator of CORT secretion. This stress test can be repeated several times, which discloses the possibility of collecting blood samples at different time points or pharmacological manipulations. There are no restrictions in terms of sex and age of the tested animal, but there are strain differences and some strains or genetic backgrounds might not react with increased activity to the described stressor.

## Author contributions

Annemarie Zimprich, Valerie Gailus-Durner, Helmut Fuchs, Martin Hrabě de Angelis, Wolfgang Wurst and Sabine M. Hölter conceived this work; Lillian Garrett, Jan M. Deussing and Carsten T. Wotjak contributed substantially to the design of the study and to the interpretation of the data. Jan M. Deussing provided CRH-COE^CNS^ and CRHR1-KO mice and was responsible for CORT measurements. Annemarie Zimprich acquired and analyzed the data; Annemarie Zimprich and Sabine M. Hölter interpreted the data and drafted the manuscript. All authors critically revised the manuscript, approved the final version to be published and agreed to be accountable for all aspects of the work in ensuring that questions related to its accuracy are appropriately investigated and resolved.

### Conflict of interest statement

The authors declare that the research was conducted in the absence of any commercial or financial relationships that could be construed as a potential conflict of interest.
