# Supplementary material for: A robust and reliable non-invasive test for stress responsivity in mice
Source: Front Behav Neurosci. 2014 Apr 15;8:125. doi: 10.3389/fnbeh.2014.00125 (PMC3995076; doi:10.3389/fnbeh.2014.00125)
Supplement: Supplementary file 2 [file DataSheet2.PDF]

		Open Field - total 20 minutes											
		Arena		Periphery				Centre					
**Cohort**	**Stress Duration**	Resting time [s]	Average Speed [m/s]	Distance travelled [cm]	Resting time [s]	Perma nence time [s]	Average Speed [m/s]	Distance travelled [cm]	Resting time [s]	Perma nence time [s]	Average Speed [m/s]	Latency [s]	Number of Entries [#]
**1-Bl6J**	15minutes	ns	ns	T↑	ns	ns	ns	ns	ns	ns	*↑	ns	ns
**2-Bl6N**	15minutes	ns	ns	ns	ns	ns	ns	ns	ns	ns	ns	ns	ns
**3-Bl6J**	50 minutes	ns	ns	ns	ns	**↑	ns	ns	*↓	**↓	*↑	ns	ns
**4-Bl6J**	50 minutes	ns	ns	ns	ns	ns	ns	ns	T↓	ns	ns	ns	ns
**5-Bl6J**	50 minutes	ns	*↑	*↑	ns	ns	*↑	ns	ns	ns	ns	ns	ns
**6-Bl6J**	2 hours	ns	*↑	**↑	ns	*↑	*↑	ns	ns	*↓	*↑	ns	ns
**7-Bl6J**	2 hours	ns	ns	ns	ns	ns	ns	ns	ns	ns	ns	ns	ns
**8-Bl6J**	2 hours	ns	ns	ns	ns	ns	ns	ns	ns	ns	ns	ns	ns
**9-Bl6N**	2 hours	ns	*↑	*↑	ns	ns	*↑	ns	ns	ns	T↑	*↑	ns
